# Acute treatment residual depression symptoms and functional impairment among depressive patients of different age groups and education levels in China: A prospective, multicenter, randomized study

**DOI:** 10.1002/brb3.70024

**Published:** 2024-09-11

**Authors:** Si Zu, Dong Wang, Jiexin Fang, Le Xiao, Xuequan Zhu, Wenyuan Wu, Xiufeng Lin, Gang Wang, Yongdong Hu

**Affiliations:** ^1^ Department of Psychiatry Beijing Chaoyang Hospital Capital Medical University Beijing China; ^2^ The National Clinical Research Center for Mental Disorders & Beijing Key Laboratory of Mental Disorders Beijing Anding Hospital, Capital Medical University Beijing China; ^3^ Department of Psychiatry Tongji Hospital of Tongji University Shanghai China; ^4^ School of Information, Renmin University Beijing, China Research Institute, Kunlun Digital Technology, Co, Ltd. Beijing, China Beijing China; ^5^ Advanced Innovation Center for Human Brain Protection Capital Medical University Beijing China

**Keywords:** age, depression, education, follow‐up, residual symptom

## Abstract

**Objective:**

A prospective, multicenter, randomized study evaluated the efficacy of major depressive disorder (MDD) patients after 2–3 months of acute treatment based on the dual factors of education and age.

**Methods:**

This study classified the included patients into four groups using two classification parameters: age (≤45 years, vs. >45 years) and education years (≤12 vs. >12). We analyzed age, gender, marital status, personal income, depression onset history, medication use, and follow‐up across various groups. We evaluated residual somatic symptoms and social functioning in depression patients was conducted using the 16‐item Quick Inventory of Depressive Symptomatology Self‐report (QIDS‐SR16), the Patient Health Questionnaire‐15 (PHQ15), and the Sheehan Disability Scale (SDS).

**Results:**

In China, 16 hospitals, 553 depression patients, and 428 fulfilled the inclusion criteria. Baseline patient data revealed significant differences among the different age groups in gender, marital status, income, first onset age, physical illness, combination of antipsychotics, and benzodiazepines use (all *p* < .05). Statistically significant differences were observed in overall comparisons among the four groups, encompassing the QIDS‐SR16 score, PHQ15 score, and various SDS parameters (all *p* < .05). However, no statistically significant differences (all *p* > .05) were found in residual somatic symptoms and social functioning parameters between different education levels (≤12 years vs. >12 years) at baseline, 3 months, and 6 months, based on total scores on the scale. Repeated measures mixed model indicates that the QIDS‐SR16 assessment indicates statistical differences among various marital statuses, income levels, medical histories, and antipsychotic medication use (*p* < .05). Furthermore, PHQ‐15 and SDS assessments reveal statistical differences between single and married/cohabiting statuses, physical comorbidities, 3 and 6 months follow‐ups compared to baseline (*p* < .05).

**Conclusion:**

This study indicates that compared to depressive patients >45 years old, those ≤45 years old often exhibit more residual depression, somatic symptoms, and severe social functional impairment; patients' education levels less influence this trend.

## Graphical abstract

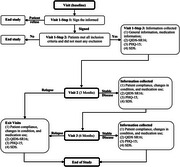

## 1 LIMITATIONS

Although this study was prospective, double‐blind, multicenter, and randomized, it still presented certain limitations: (1) Lack of detailed information: while the study assessed patients' education duration, it did not delve deeper into the specifics of their occupational roles. Varied occupations might impact symptom remission in depressed patients. Additionally, the choice of different antidepressants and the participation of depressed patients in group activities or receiving therapeutic support from their families can significantly influence symptom remission. These factors could potentially affect the treatment of depressed patients. (2) Reliance on self‐assessment scales: the study employed self‐assessment scales to evaluate patients' depression. Although these self‐rated assessments reflect patients' symptoms and emotions, they might not offer an entirely objective evaluation due to patients' limited comprehension of scale information. This potential bias could be mitigated by supplementing with assessments from other sources. (3) Observational nature of the study: as an observational study, the results do not establish causality between variables, highlighting the need for further research to ascertain causal relationships. Due to the inherent trade‐offs in the study, the handling of age can also affect the outcome of the results. (4) In addition, research on confounding factors related to residual depressive symptoms that may result from depression is still insufficient. For example, a study (Pitanupong & Aunjitsakul, 2023) on patients with severe depression showed that individuals with residual depressive symptoms had higher neuroticism scores and lower conscientiousness scores. This is also an aspect that needs attention in our future research.

## 2 INTRODUCTION

Depression is characterized by enduring and pronounced depression and a loss of interest, making it the most prevalent psychological disorder in contemporary society (Malgaroli et al., 2021). The prevalence of depression fluctuates across countries and regions, yet it has exhibited an upward trend in recent years across most geographical areas (Shin et al., 2021; Snippe et al., 2021). Statistics indicate that the prevalence of depression was approximately 2.1% in China (Lu et al., 2021).

The primary symptoms of depression encompass enduring feelings of joylessness and diminished volitional behavior (≥2 weeks). These symptoms often include inappropriate guilt, suicidal thoughts, difficulties in concentration, insomnia, and appetite disturbances. Patients with depression frequently exhibit a notable inclination toward recurrence or chronic. Approximately 50% or fewer of those experiencing a first‐episode encounter a recurrence within the subsequent 5 years, often accompanied by suicidal thoughts and behaviors. Eventually, over 15% of these patients may tragically succumb to suicide (Rotenstein et al., 2016). The 2019 outbreak of COVID‐19 resulted in a surge of home isolation worldwide. This situation has contributed to heightened stress levels, disrupted sleep patterns, and an uptick in the prevalence of anxiety and depression across the general population (Mazza et al., 2020). In a study involving 1653 participants, over 70% reported experiencing moderate or higher stress levels. Among them, 59% met the criteria for clinically significant anxiety, while 39% exhibited moderate depressive symptoms (Varma et al., 2021). Pitanupong and Aunjitsakul (2023) study indicates that residual symptoms of depression can have a significantly negative impact on patient's daily lives, leading to psychological imbalance and an increase in stigma. The heightened stigma, in turn, exacerbates the depressive symptoms in individuals with major depressive disorder (MDD), creating a vicious cycle.

The aim of treating depression is achieving complete symptom remission and functional recovery, which means the elimination of all depressive symptoms and the restoration of the patient's psychosocial functioning to their state before the onset of the illness. However, a substantial portion of patients continue to experience residual symptoms following acute depression treatment (Pitanupong & Sammathit, 2023). Residual symptoms, also known as subthreshold depressive symptoms, when a patient has not fully recovered despite a reduction in their symptoms (Wang et al., 2020).

Studies indicate that prevalent residual symptoms of depression, such as sleep disturbances, fatigue, somatic symptoms, and cognitive dysfunction, can significantly affect patients (Conradi et al., 2011; McClintock et al., 2011). The lingering functional impairment observed in certain patients after the acute treatment phase can endure for an extended period. Both residual symptoms and functional impairment substantially elevate the risk of depression relapse and recurrence (Harkness et al., 2014). Investigating the characteristics of residual symptoms and their influencing factors can aid in crafting more precise treatment strategies for depression. This approach can facilitate symptoms alleviation in patients and diminish the likelihood of relapse and recurrence.

The World Health Organization (WHO) reported a gradual increase in the prevalence of depression within the population as age increasing, according to their 2019 findings (Hofmann, 2020). A study conducted in Pakistan revealed that the likelihood of depressive disorder among young rural women was approximately 4.4% (Rahman et al., 2009). However, in the Baltimore, Maryland, metropolitan area, the prevalence of MDD among African Americans aged 19–22 years was 9.4% (Ialongo et al., 2004). Another study indicated that the prevalence of depression among the elderly population in Asia ranged from approximately 7.8%−46% (Mohd et al., 2019). The studies above suggest that age may be a crucial factor influencing the onset of depression. However, there have been fewer studies investigating age‐related factors concerning residual symptoms and functional impairment following acute depression treatment.

There is widespread recognition of education's significant role in depression. Numerous studies have explored the correlation between education and depressive symptom. For instance, Bauldry (2015) discovered that women with diabetes exhibited more depressive symptoms as their education level decreased. Michael's study on American young adults revealed that individuals holding a college degree displayed lower levels of depressive symptoms compared to those with a high school degree or less (McFarland & Wagner, 2015). Nonetheless, a study conducted among individuals aged over 45 years in China demonstrated variations in the prevalence of depression among groups with differing levels of education (Bi et al., 2021). The above studies investigated the correlation between educational attainment and depression. However, they did not further investigate the correlation between residual symptoms and educational attainment during the recovery process of individuals with depression. The study involving Chinese patients with depression did not undertake a comparative recovery analysis encompassing residual symptoms of depression, somatic symptoms, and functional impairment among patients across various age and educational backgrounds. Patients with depression stand to gain the most from a comprehensive and personalized treatment approach. Consequently, when formulating treatment plans for each patient, factors such as pathological and pharmacological mechanisms, age, education, income, and occupation should be incorporated into the study for a more holistic understanding. Analyzing residual symptoms and social functioning among depressed patient groups categorized by age and educational background will offer insights into the characteristics of symptom alterations throughout the recovery process of different depression cohorts. This understanding will enable the development of more tailored and effective treatment strategies.

Furthermore, due to the scarcity of extensive, multicenter participatory reports tracking symptoms post‐acute‐phase depression treatment, there is significant interest in filling this gap. Addressing this, an examination of the impact of age and educational attainment on outcomes among depressed patients through a multicenter, observational investigation in real‐world clinical settings in China is warranted. The aim of this study were twofold: (1) to compare residual depressive and somatic symptoms and functional impairment among depressive patients across different age groups following acute phase treatment, and (2) to compare these symptoms among patients with varying educational backgrounds within different age brackets diagnosed with MDD during the consolidation phase. This comparison aimed to comprehend the nature and frequency of residual symptoms, their evolution post‐acute treatment, and their influence on patients' social functioning.

## 3 METHODS

### 3.1 Patients collected

This study was carried out across 16 hospitals in China, including 7 specialized in psychiatric care and 9 general hospitals equipped with psychiatry/psychology departments. A cohort of 553 outpatients participate in this prospective exploratory study. The projected sample size was determined with careful consideration of the study's practicality. Patients were recruited using a continuous sampling method and were collected between June 2016 and February 2017. The study underwent thorough review and received approval from the Ethics Committee of Beijing Anding Hospital, Beijing Chaoyang Hospital, and Tongjing Hospital of Tongji University. With a complete description of the purpose and methods of this study, we obtained informed consent signed by the patient.

### 3.2 Inclusion criteria and exclusion criteria

Inclusion criteria include the following: (1) patient age ≥18 years. (2) Patient meets the diagnosis of International Classification of diseases‐10 (ICD‐10) (Quan et al., 2005) depressive episode (F32) or recurrent depressive disorder (F33). (3) The patient believes, as assessed by a visual analogue scale (VAS), that he or she has recovered 50% or more of his or her current depression compared to the beginning of the current episode. (4) The antidepressant is the patient's primary treatment medication, as determined by the psychiatrist. The categories of antidepressant medications used in this study include tricyclic antidepressants, tetracyclic antidepressants, selective serotonin reuptake inhibitors (SSRIs), serotonin‐norepinephrine reuptake inhibitors (SNRIs), noradrenergic and specific serotonergic antidepressants (NaSSAs), norepinephrine–dopamine reuptake inhibitors (NDRIs, such as bupropion), SARIs (such as trazodone and nefazodone), α2‐adrenergic receptor antagonists, 5‐Hydroxytryptamine 1 (5‐HT1) and 5‐HT2 receptor antagonists (such as mianserin), norepinephrine reuptake inhibitors (NARIs, such as reboxetine), and agomelatine, among others. (5) The patient has been treated with antidepressants for 8 weeks to 12 weeks since the current depressive episode, with no more than 2 weeks of cumulative interruptions in treatment. (6) The patient's literacy level and ability to read and understand did not affect the patient's accuracy and speed in completing the self‐assessment scale. (7) The patients were followed up for at least 6 months.

Exclusion criteria are as follows: (1) a clear previous history of manic or hypomanic episodes, or a diagnosis of bipolar disorder, schizophrenia, schizoaffective psychosis, or psychotic disorders associated with other disorders; and (2) the patients who cannot follow the study protocol based on the investigator's judgment, which includes (a) patients with evidence of drug dependence and use of psychoactive substances and (b) patients who were currently experiencing severe physical illnesses.

### 3.3 Grouping

In this study, patients were categorized into two age groups: ≤45 years and >45 years (45 is considered the dividing age between young and middle‐aged or elderly individuals) (Bi et al., 2021). Each age group has been further subdivided into two subgroups based on variations in patients' years of education: ≤12 years and >12 years (with 12 years as the delineation for primary and higher education).

### 3.4 Withdrawal from the study

Apart from the baseline follow‐up, it is crucial for investigators to actively inquire about any changes in the patient's condition since their last follow‐up visit. Patients are considered to be experiencing a relapse/recurrence when any of the following occur: (1) outpatients are hospitalized due to an exacerbation of their condition (depression); (2) patients are experiencing suicidal attempts or behaviors; or (3) in the physician judgment, the patient's clinical presentation has met the diagnostic criteria for a relapse of depression. The investigator should make every effort to reach out to the patient regarding their clinical status, urging them to visit the study center for a follow‐up as soon as possible. Additionally, it is essential to ensure the complete of the last visit, record pertinent information, and collect all self‐assessment scales before the patient withdraws from the study.

**FIGURE 1:**
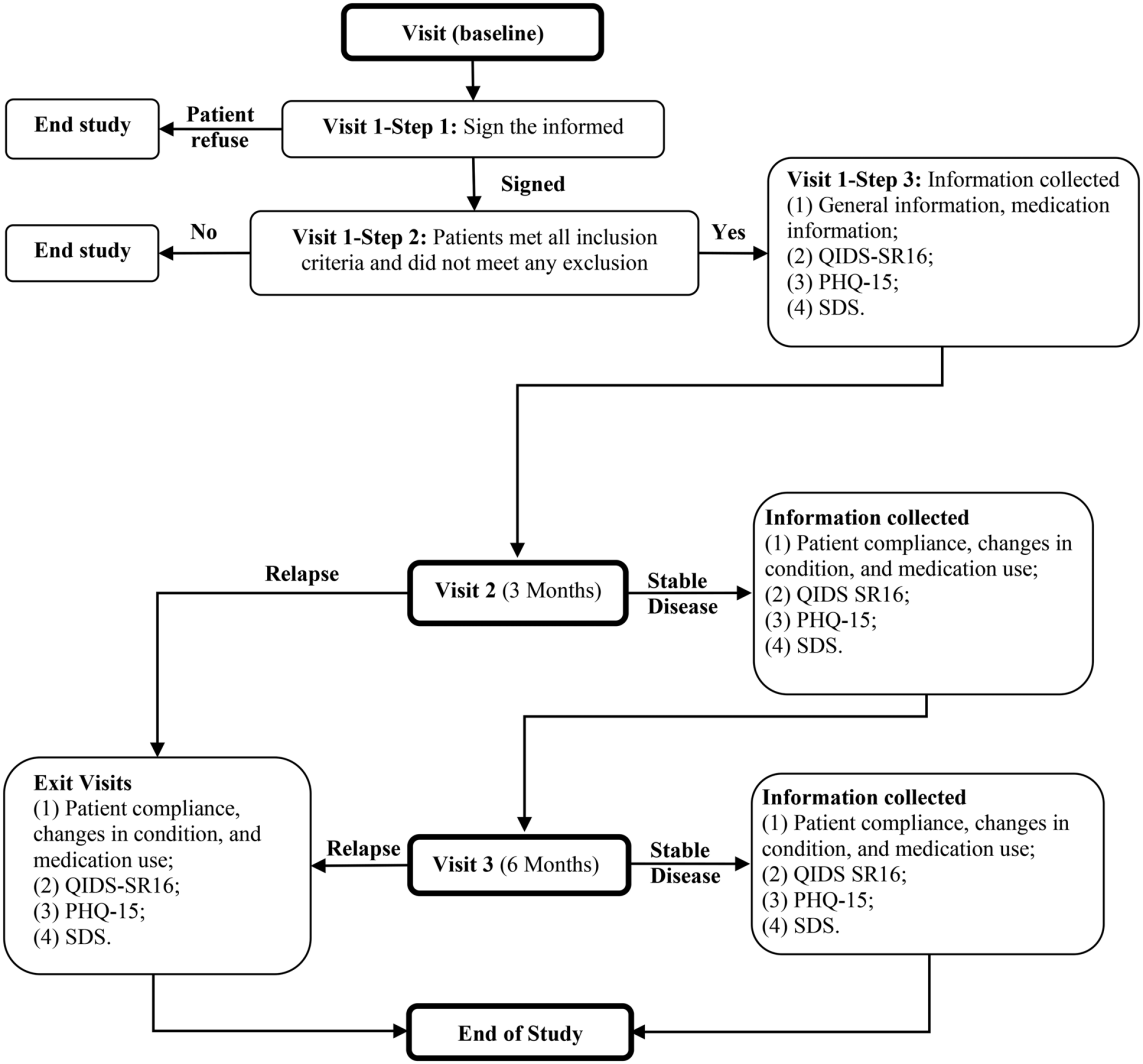
The flow chart for study design.

During the study, if the investigator identified that a patient did not meet any of the enrollment criteria, a final visit was conducted with the patient to conclude their participation in the study. Patient data was recorded, followed by the completion of all self‐assessment scales, including cognitive measures, after which they were withdrawn from the study. The above scenarios include the following: (1) patient appears to turn irritable; (2) the patient changes his or her diagnosis based on the investigator's judgment (the change in diagnosis affects the patient's eligibility for the study); and (3) the patient develops other conditions that affect his or her eligibility for the study. If a patient requests or needs to withdraw from the study for other reasons, the investigator should facilitate the patient's to completion of the withdrawal visit, if possible. The detailed study process is described in Figure 1.

### 3.5 Assessment tools

The study employed three self‐assessment scales to comprehensively evaluate patients' depressive state, somatic symptoms, and the impact of depressive symptoms on their functioning at home, work/school, and socially settings, respectively. (1) Brief 16‐item Quick Inventory of Depressive Symptomatology Self‐Report (QIDS‐SR16) to assess patients' depressive symptoms (Rush et al., 2006). The QIDS‐SR16 comprises 16 items, scored on a 4‐point scale ranging from 0 to 3, resulting in a total score of 27. Higher scores indicate a more severe depressive condition. A QIDS‐SR16 score > 5 signifies the presence of residual symptoms during the follow‐up period.

Patient Health Questionnaire‐15 (PHQ‐15) consists of 15 items to assess the severity of the patient's somatic symptoms (Ran et al., 2020). The somatic symptoms include pain in stomach, back, arms, legs, head, chest or during sexual intercourse, menstrual cramps, dizziness, fainting spells, feeling one's heart pound, shortness of breath, nausea, gas or indigestion, feeling tired or having low energy and trouble sleeping. The scale is scored on a 0–2 scale with a 3‐point scale, and the total score ranges from 0 to 30. A higher score on the PHQ‐15 means severe somatic symptoms. (3) The Sheehan disability scale (SDS) scale consists of 3 items that measure the impact of depressive symptoms on the individual's performance at home, work/school, and socially (Jokelainen et al., 2019). The scale uses a 0–10 scale with a total score of 0–30. High SDS scores indicate the presence of more significant functional impairment. (4) The VAS is widely used to assess the degree of pain in patients (Domeshek et al., 2017). In this study, a VAS is employed to gauge the degree of recovery from depression in patients. Patients indicate their assessment by marking a paper line from 0 to 10. The scale of 0 to 10 delineates various recovery levels, with “0” indicating the most severe state and “10” indicating complete recovery. Patients selecting a rating of 5 or higher align with the inclusion criteria, signifying that they perceive their depression to have improved by 50% or more.

### 3.6 Study processes

Before the clinical study commenced, a collective training session was conducted for the principal investigators at each research center. The training encompassed different elements of the study protocol, such as diagnostic criteria, creating case report forms (CRFs), and operational procedures. We performed a 6‐month follow‐up on patients evaluated at the outset and the third and sixth months, utilizing diverse scales (Figure 1).

The data to be collected encompassed baseline patient details, medication usage, disease traits, and scores derived from the QIDS‐SR16, PHQ15, and SDS. Throughout the follow‐up, if a patient's status remained unchanged, they proceeded to the subsequent observational period. Patients meeting any conditions detailed in Section 3.4 withdrawal from the study. Independent evaluators carried out assessments using the scales. This study did not involve any treatment interventions; all therapies for the present ailment were permissible.

### 3.7 Statistical analysis

All data in this study were statistically analyzed using SPSS 25.0. The chi‐square test was employed to compare count data. For measurement data conforming to the chi‐square distribution, pairwise comparisons were conducted using the *t*‐test. Nonparametric tests were utilized to calculate measurement data not adhering to the chi‐square distribution. Cronbach's α coefficient was used to assess the measurement reliability of various scales related to depression. The effect of age or education on changes of QIDS‐SR16, PHQ15, and SDS from baseline were examined using multivariate linear mixed model for repeated measures respectively, including visits (month 3, month 6), education, age as fixed effect, subject as random effect, and marital status, income, disease feature as covariates. A significance level of *p* < .05 was deemed statistically significant across all statistical methodologies.

## 4 RESULTS

### 4.1 Sample characteristics

**FIGURE 2:**
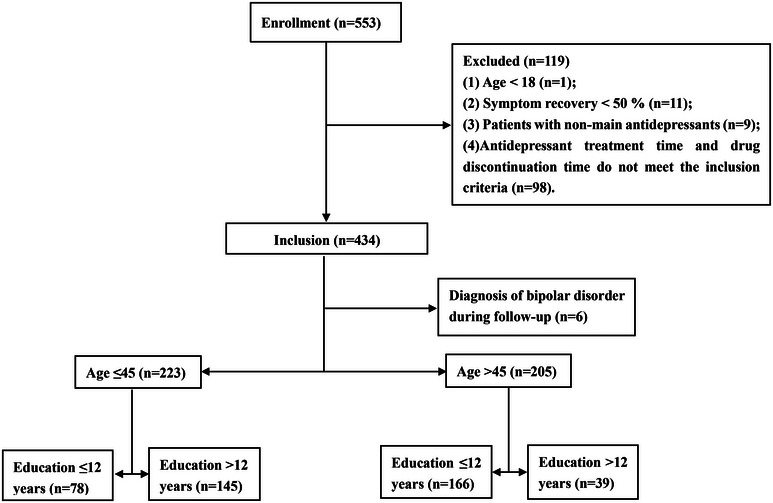
The flow diagram of patient enrollment.

In this study, a total of 553 patients with depression were initially screened, and 119 patients who did not meet the inclusion criteria were subsequently excluded. Subsequently, 434 patients were followed up for observation. During this period, 6 cases exhibited transient mania, leading to a diagnosis change to bipolar disorder, and they were consequently excluded from the analysis. Finally, 428 patients met the inclusion criteria and underwent further analysis. By the end of the sixth month, 110 patients were lost to follow‐up and thus dropped from the study. Detailed results are shown in **Figure** **2**.

### 4.2 Analysis of patients' baseline data

This study included 223 participants aged ≤45 years and 205 participants aged >45 years. Among the age group of ≤45 years old, there were 78 patients (35.0%) with ≤12 years of education and 145 patients (65.0%) with >12 years of education. In the age group of >45 years old, there were 166 patients (81.0%) with ≤12 years of education and 39 patients (19.0%) with >12 years of education.

An analysis of the baseline data revealed significant statistical differences between different age groups (≤45 years old vs. >45 years old ) concerning gender, marital status, income, age of the initial symptoms, physical illness, use of antipsychotics and benzodiazepines combinations, as well as the scores on the QIDS‐SR16, PHQ15, and SDS scales (all *p* < .05) (Table 1).

**TABLE 1:** Baseline demographics and disease characteristics.

Age	≤45 years (*N* = 223)		> 45 years (*N* = 205)		Statistic	*p*‐value △
Education years	≤12 years *N* = 78	>12 years *N* = 145	≤12 years *N* = 166	>12 years *N* = 39		
**Baseline**						
Age	35.6 (7.5)	31.2 (6.6)	57.9 (7.4)	54.3 (7.4)	–	–
Gender					6.64	.003
Male	29 (37.2)	56 (38.6)	38 (22.9)	13 (33.3)		
Female	49 (62.8)	89(61.4)	128 (77.1)	26 (66.7)		
Marital status					92.68	<.001
Married/cohabit	59 (76.6)	77 (53.1)*	147 (89.1)	34 (87.2)		
Divorced/separate/widowed	3 (3.9)	7 (4.8)	17 (10.3)	5 (13.0)		
Single	15 (19.5)	61 (42.1)	1 (0.6)	0 (0)		
**Income**					25.48	<.001
≤1000¥	21 (26.9)	31 (21.4)*	45 (27.4)	2 (5.1)*		
1000–5000¥	46 (59.0)	52 (35.9)	106 (64.6)	22 (56.4)		
≥5000¥	7 (9.0)	38 (26.2)	10 (6.1)	12 (30.8)		
≥10,000¥	4 (5.1)	24 (16.5)	3 (1.8)	3 (7.7)		
**Disease feature**						
Age of first onset	35.0 (25.9, 40.4)	29.7 (24.4, 34.7)*	55.0 (48.7, 60.4)	49.1 (46.0, 59.0)*	270.4	<.001
First episode patients	41 (52.6)	98 (67.6)*	101 (60.8)	21 (53.9)	0.36	.550
Physical illness	13 (16.7)	17 (11.7)	82 (49.4)	13 (33.3)	55.88	<.001
Family history of mental illness	6 (8.7)	17 (13.8)	14 (9.7)	74 (22.6)	<0.001	1.00
Onset time (weeks)	16 (10, 24)	14.5 (10, 24.5)	16 (10, 26)	12 (9, 24)	0.010	.926
**Treatment characteristics**						
Mono‐antidepressant therapy	56 (71.8)	113 (77.9)	111 (66.9)	29 (74.4)	2.99	.084
≥2 antidepressant	22 (28.2)	32 (22.1)	55 (33.1)	10 (25.6)		
Combination of antipsychotics	28 (35.9)	43 (29.7)	76 (45.8)	15 (38.5)	7.15	.008
Changing antidepressants	11 (14.1)	13 (9.0)	23 (13.9)	7 (18.0)	1.45	.228
Benzodiazepines	48 (61.5)	64 (44.1)*	102 (61.6)	22 (56.4)	4.55	.033
**Evaluation index**						
QIDS‐SR16 score	7.7 (4.9)	8.1 (4.7)	6.4 (4.3)	7.3 (4.0)	3.18	.002
PHQ15 score	5.6 (4.9)	6.6 (4.1)	5.5 (4.5)	5.3 (4.0)	2.67	.008
SDS score	8.8 (7.2)	8.5 (6.0)	6.0 (6.5)	8.1 (8.1)	19.67※	<.001
SDS: work/study	3.5 (3.0)	3.0 (2.3)	2.3 (2.7)	2.8 (2.9)	15.33※	<.001
SDS: social life	2.9 (2.6)	2.9 (2.3)	2.2 (2.6)	3.1 (3. 1)	12.87※	<.001
SDS: family responsibility	2.7 (2.5)	2.7 (2.4)	2.0 (2.6)	2.6 (3.0)	11.80※	<.001
**Residual symptoms**						
QIDS‐SR16 > 5	48 (61.5%)	97 (66.9%)	81 (48.8%)	23 (59.0%)	8.97※	.028

The comparison between the two groups of education duration (≤ 2 years and > 12 years) is marked with an asterisk (*) for statistically significant differences, while a circle (※) indicates non‐parametric tests.

Among patients aged ≤45 years, significant differences were observed in marital status, income, age of the first onset, the occurrence of the first episode, and the use of benzodiazepines among patients with varying education levels (all *p* < .05). However, no significant differences were found in the scores on the scales across these education levels (all *p* > .05).

Among patients over 45, significant differences were observed only in income and age of the first onset (*p* < .05). There were no statistically significant differences in other demographic characteristics and scale scores (*p* > .05) among patients with different education levels (Table 1).

### 4.3 Residual symptoms

Patients with residual symptoms after acute treatment were derived based on QIDS‐SR16 > 5. Table 1 illustrates that among patients aged ≤45 years, 48 patients (61.5%) with education ≤12 years and 97 patients (66.9%) with education >12 years; among patients >45 years, 81 patients (48.8%) with education ≤12 years and 23 patients (59.0%) with education >12 years. Education did not influence residual symptom prevalence in patients aged ≤45 years and >45 years; nonetheless, those ≤45 years had significantly more symptoms than those >45 years (*p* = .028).

### 4.4 PHQ‐15 and QIDS‐SR16 scale

**FIGURE 3:**
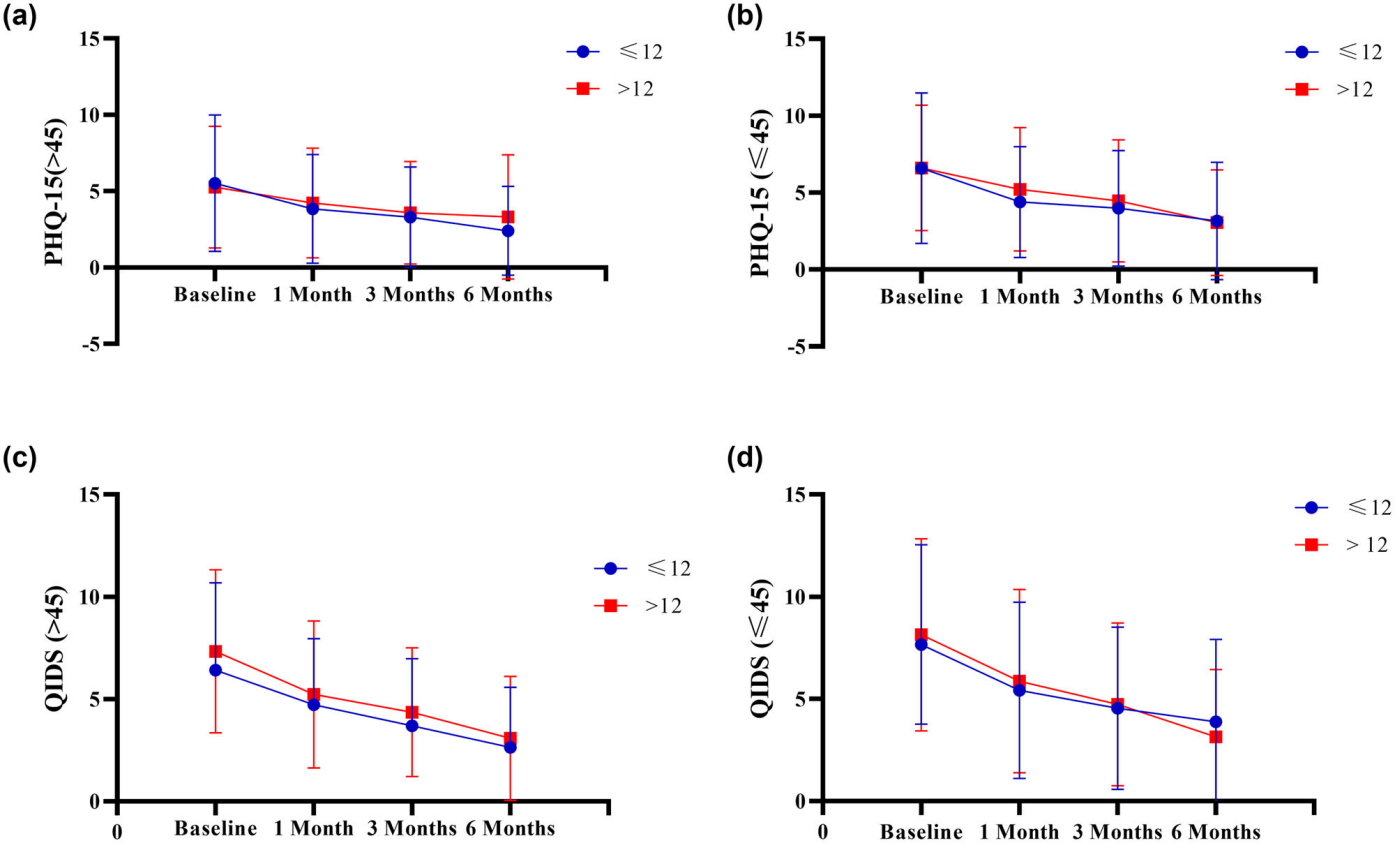
**The self‐measurement scale for PHQ‐15 and QIDS after 6 months of follow‐up**. A: Comparison of patients at age > 45 years with different levels of education (≤12 vs. > 12) for PHQ‐15. B: Comparison of patients at age ≤45 years with different levels of education (≤12 vs. > 12) for PHQ‐15. C: Comparison of patients at age > 45 years with different levels of education (≤12 vs. > 12) for QIDS. D: Comparison of patients at age ≤45 years with different levels of education (≤12 vs. > 12) for QIDS.

In the patient group aged >45 years, the PHQ‐15 scores and the QIDS‐SR16 scores did not show statistical differences between the subgroups with ≤12 and >12 years of education (*p* > .05) at 3 and 6 months post‐acute treatment (Table 2); the trends across various follow‐up periods are depicted in Figure 3a and c. Similarly, within the group of patients aged ≤45 years, the PHQ‐15 scores and QIDS‐SR16 scores did not exhibit statistical differences between subgroups with ≤12 and > 12 years of education (*p* > .05) (Table 3); the trends across different follow‐up points are presented in Figure 3b and d.

**TABLE 2:** Total scores on each scale at baseline, 3 months, and 6 months for patients with different levels of education in the age >45 years old group.

Scale/age	≤12 years	>12 years	*t*/*Z*	*p*
**Baseline**				
QIDS‐SR16 score	6.41 (4.28)	7.33 (3.98)	2.1111	.146
PHQ15 score	5.52 (4.46)	5.26 (3.98)	0.0420	.838
SDS score	5.96 (6.48)	8.1 (8.11)	1.5207	.218
SDS: work/study	2.25 (2.74)	2.76 (2.87)	0.9299	.335
SDS: social life	2.15 (2.62)	3.13 (3.12)	3.5367	.060
SDS: family responsibility	2.04 (2.55)	2.64 (3)	1.4392	.230
**Three months**				
QIDS‐SR16 score	3.7 (3.27)	4.35(3.15)	1.6986	.193
PHQ15 score	3.3 (3.29)	3.58 (3.35)	0.2393	.625
SDS score	2.8 (4.47)	2.97 (3.86)	1.0377	.308
SDS: work/study	1.08 (1.81)	0.86 (1.27)	0.0556	.814
SDS: social life	0.94 (1.63)	1.27 (2.05)	0.7823	.376
SDS: family responsibility	0.91 (1.53)	0.9 (1.56)	0.0579	.810
**Six months**				
QIDS‐SR16 score	2.64 (2.93)	3.08 (3.03)	0.5054	.477
PHQ15 score	2.4 (2.91)	3.32 (4.07)	1.4411	.230
SDS score	2.28 (4.28)	3.36 (5.38)	0.5578	.455
SDS: work/study	0.84 (1.57)	0.91 (1.76)	0.1713	.679
SDS: social life	0.82 (1.79)	1.42 (2.24)	1.9185	.166
SDS: family responsibility	0.69 (1.46)	1.16 (2.21)	1.0336	.309

**TABLE 3:** Total scores on each scale at baseline, 3 months, and 6 months for patients with different levels of education in the age ≤45 years old group.

Scale/age	≤12 years	>12 years	*t*/*Z*	*p*
**Baseline**				
QIDS‐SR16 score	7.65 (4.89)	8.14 (4.7)	0.5586	.455
PHQ15 score	6.59 (4.89)	6.61 (4.08)	0.1327	.716
SDS score	8.79 (7.17)	8.48 (5.95)	0.0183	.892
SDS: work/study	3.52 (2.99)	3.02 (2.34)	0.7039	.402
SDS: social life	2.95 (2.56)	2.92 (2.32)	0.0278	.868
SDS: family responsibility	2.74 (2.55)	2.71 (2.37)	0.0488	.825
**Three months**				
QIDS‐SR16 score	4.54 (3.97)	4.74 (3.98)	0.0950	.758
PHQ15 score	3.98 (3.76)	4.47 (3.97)	0.5903	.442
SDS score	5.11 (6.11)	4.29 (5.16)	0.2912	.589
SDS: work/study	2.05 (2.45)	1.59 (1.96)	0.7318	.392
SDS: social life	1.68 (2.05)	1.29 (1.81)	1.9257	.165
SDS: family responsibility	1.51 (2.02)	1.41 (1.94)	0.0510	.821
**Six months**				
QIDS‐SR16 score	3.88 (4.04)	3.15 (3.29)	0.5387	.463
PHQ15 score	3.16 (3.81)	3.06 (3.43)	0.0205	.886
SDS score	4.44 (6.2)	2.72 (4.07)	1.6038	.205
SDS: work/study	1.72 (2.51)	0.97 (1.4)	1.2721	.259
SDS: social life	1.46 (2.02)	0.86 (1.46)	3.0990	.078
SDS: family responsibility	1.39 (2.07)	0.89 (1.7)	2.1739	.140

### 4.5 SDS detection

**FIGURE 4:**
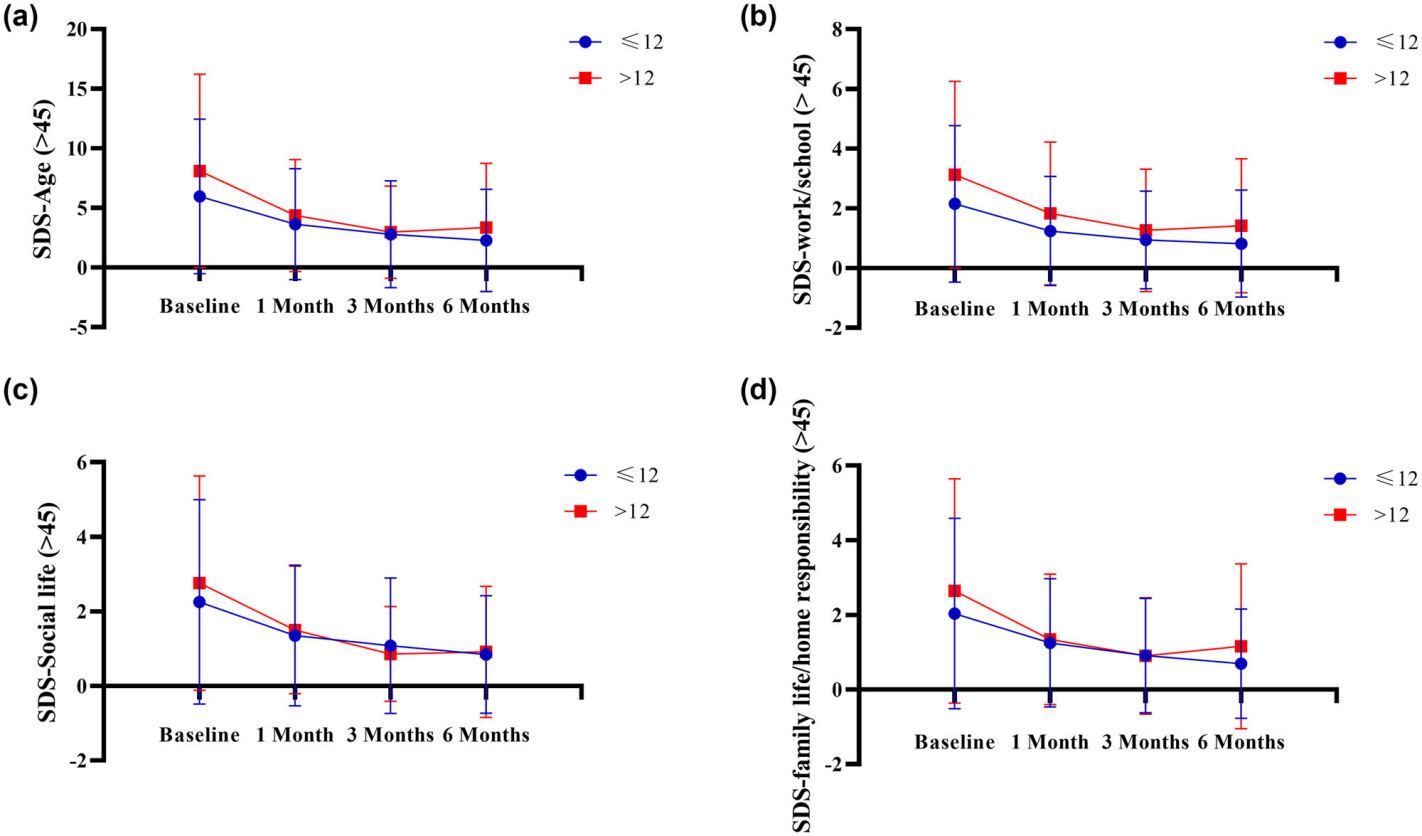
**The self‐measurement scale for SDS after 6 months of follow‐up for age >** **45 years**. A: Comparison of patients with different levels of education (≤12 vs. > 12) for SDS‐age. B: Comparison of patients with different levels of education (≤12 vs. > 12) for SDS work/school. C: Comparison of patients with different levels of education (≤12 vs. > 12) for SDS social life. D: Comparison of patients with different levels of education (≤12 vs. > 12) for SDS family life/home responsibility.

**FIGURE 5:**
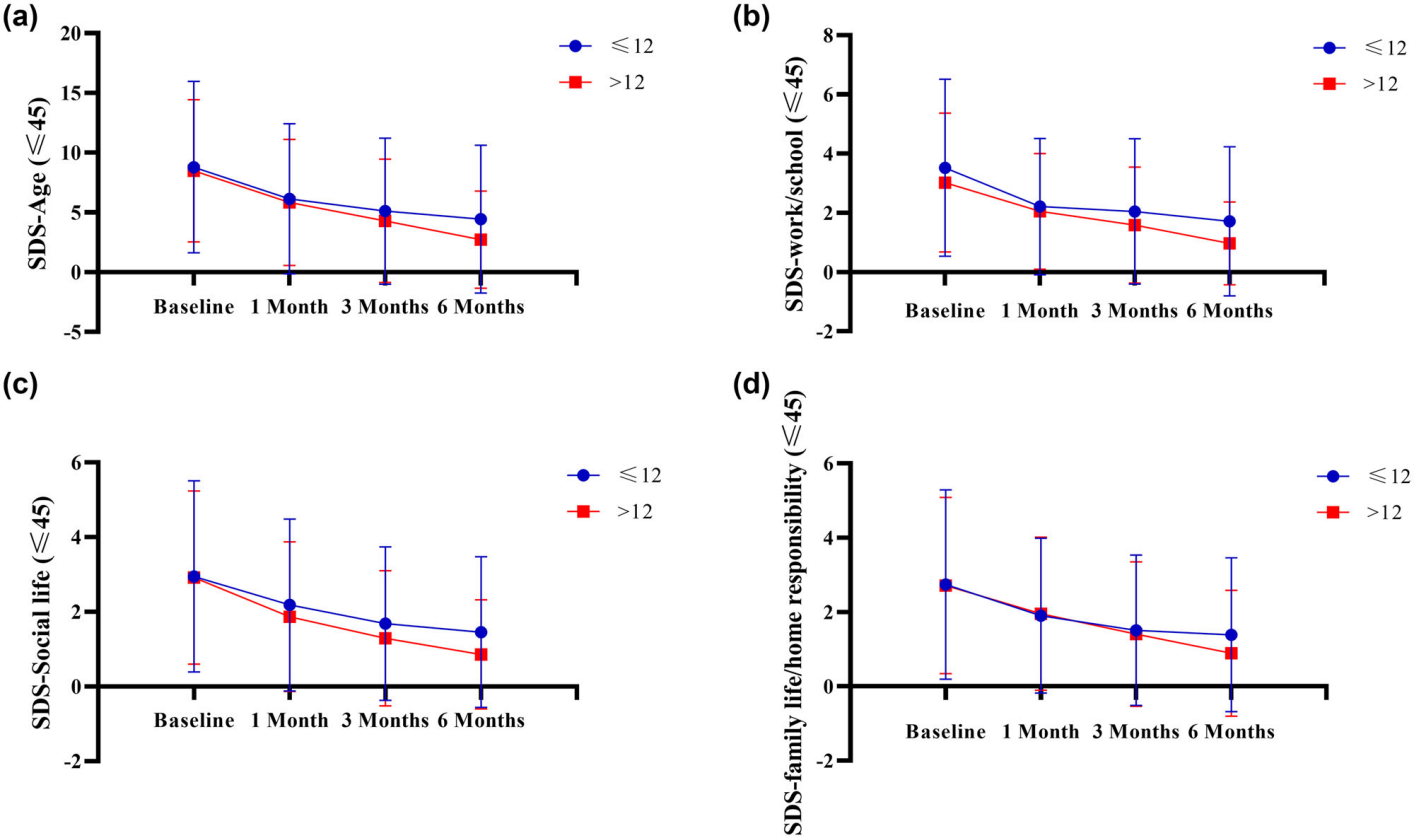
**The self‐measurement scale for SDS after 6 months of follow‐up for age ≤45 years**. A: Comparison of patients with different levels of education (≤12 vs. > 12) for SDS‐age. B: Comparison of patients with different levels of education (≤12 vs. > 12) for SDS work/school. C: Comparison of patients with different levels of education (≤12 vs. > 12) for SDS social life. D: Comparison of patients with different levels of education (≤12 vs. > 12) for SDS family life/home responsibility.

In the groups aged over 45 (Figure 4 and Table 2) and ≤45 years (Figure 5 and Table 3), there were no statistically significant differences (*p* > .05) between patients with different education levels (≤12 years vs. >12 years) regarding the SDS total score, domestic work/school study, social life, and family responsibility.

### 4.6 Mixed model for repeated measures

We used a mixed model for repeated measures (MMRM) to examine the impact of age or educational level on the score of the QIDS‐16, PHQ15, and SDS, including covariates identified through multivariate linear mixed model.

The results of the QIDS‐SR16 assessment show statistical differences between different marital statuses (Divorced/Separated or Single vs. Married/Cohabit) and income levels (≥5000¥ vs. ≤1000¥), all with a significance level of *p* < .05. There are also statistical differences in patients with physical comorbidity and the use of a combination of antipsychotics (all *p* < .05). Recurrence rates and follow‐up (3 or 6 months vs. baseline) results also exhibit statistical differences (all *p* < .05). However, no statistical differences are observed among the remaining indicators (all *p* > .05) (Table 4).

**TABLE 4:** Repeated measure analyses predicting score of QIDS‐SR16.

Variables	Groups	Beta	SE	*Z*	*p*
Intercept		8.076	0.486	16.62	<.001
Education years	≤12 years				
	>12 years	0.492	0.254	1.94	.054
Age	≤45 years				
	> 45 years	−0.805	0.262	−3.08	.002
Follow‐up	Baseline				
	Month 3	−3.017	0.282	−10.69	<.001
	Month 6	−4.099	0.286	−14.36	<.001
Gender	Male				
	Female	−0.402	0.235	−1.71	.088
Marital status	Married/cohabit				
	Divorced/separate	1.863	0.561	3.32	.001
	Widowed	0.507	0.492	1.03	.304
	Single	1.096	0.315	3.48	.001
Income	≤1000¥				
	1000–5000¥	−0.214	0.266	−0.8	.422
	≥5000¥	−1.127	0.355	−3.17	.002
	≥10,000¥	−0.157	0.498	−0.31	.753
Recurrence	Recurrence				
	First episode	−0.886	0.216	−4.11	<.001
Physical comorbidity	No				
	Yes	0.504	0.244	2.07	.039
Family history of mental illness	No				
	Yes	0.207	0.318	0.65	.517
Mono‐antidepressant	No				
	Yes	−0.183	0.234	−0.78	.435
Combination of antipsychotics	No				
	Yes	0.458	0.212	2.16	.031
Benzodiazepines	No				
	Yes	−0.287	0.212	−1.35	.177

The patient's PHQ‐15 assessment results demonstrate statistical differences between Single and Married/Cohabit statuses and the occurrence of Physical comorbidity (*p* < .05). The PHQ‐15 values at three and 6 months of follow‐up show statistical differences compared to baseline (*p* < .05). No statistical differences are observed among the remaining indicators (all *p* > .05) (Table 5).

**TABLE 5:** Repeated measure analyses predicting score of PHQ‐15.

Variables	Groups	Beta	SE	*Z*	*p*
Intercept		5.422	0.504	10.76	<.001
Education years	≤12 years				
	>12 years	0.327	0.264	1.24	.217
Age	≤45 years				
	> 45 years	−1.089	0.272	−4	<.001
Follow‐up	Baseline				
	Month 3	−2.163	0.292	−7.4	<.001
	Month 6	−3.168	0.297	−10.69	<.001
Gender	Male				
	Female	0.428	0.244	1.76	.078
Marital status	Married/cohabit				
	Divorced/separate	0.233	0.587	0.4	.692
	Widowed	−0.12	0.510	−0.2	.842
	Single	0.862	0.327	2.63	.009
Income	≤1000¥				
	1000‐5000¥	0.404	0.275	1.47	.143
	≥5000¥	−0.282	0.368	−0.77	.445
	≥10,000¥	0.378	0.519	0.73	.467
Recurrence	Recurrence				
	First episode	−0.208	0.224	−0.93	.352
Physical comorbidity	No				
	Yes	0.821	0.254	3.23	.001
Family history of mental illness	No				
	Yes	−0.322	0.332	−0.97	.332
Mono‐antidepressant	No				
	Yes	0.181	0.242	0.75	.454
Combination of antipsychotics	No				
	Yes	0.371	0.220	1.69	.092
Benzodiazepines	No				
	Yes	−0.221	0.220	−1.01	.315

The patient's SDS assessment results indicate statistical differences between genders, Single and Married/Cohabit statuses, and Physical comorbidity (all *p* < .05). Recurrence rates and follow‐up (3 or 6 months vs. baseline) show statistical differences in SDS assessment (*p* < .05). However, no statistical differences are observed among the remaining indicators (all *p* > .05) (Table 6).

**TABLE 6:** Repeated measure analyses predicting score of SDS.

Variables	Groups	Beta	SE	*Z*	*p*
Intercept		8.884	0.698	12.72	<.001
Education years	≤12 years				
	>12 years	0.422	0.366	1.15	.250
Age	≤45 years				
	> 45 years	−1.708	0.377	−4.53	<.001
Follow‐up	Baseline				
	Month 3	−3.999	0.405	−9.87	<.001
	Month 6	−4.766	0.411	−11.61	<.001
Gender	Male				
	Female	−1.190	0.337	−3.53	.001
Marital status	Married/cohabit				
	Divorced/separate	1.224	0.813	1.51	.133
	Widowed	0.534	0.7059	0.76	.450
	Single	1.348	0.452	2.98	.003
Income	≤1000¥				
	1000‐5000¥	−0.369	0.381	−0.97	.334
	≥5000¥	−1.582	0.510	−3.1	.002
	≥10,000¥	−1.032	0.714	−1.44	.150
Recurrence	Recurrence				
	First episode	−1.040	0.309	−3.36	.001
Physical comorbidity	No				
	Yes	0.804	0.350	2.3	.022
Family history of mental illness	No				
	Yes	0.1701	0.459	0.37	.711
Mono‐antidepressant	No				
	Yes	0.580	0.336	1.73	.085
Combination of antipsychotics	No				
	Yes	0.096	0.304	0.32	.753
Benzodiazepines	No				
	Yes	0.201	0.304	0.66	.510

### 4.7 The validity of the QIDS‐SR16, PHQ‐15, and SDS

We used Cronbach's α coefficient to assess the measurement reliability of various scales related to depression. When Cronbach's α coefficient is > 0.8, it is considered that the reliability of the measurement indicator data is excellent. The results indicated that the Cronbach's α values for QIDS‐SR16, PHQ‐15, and SDS were 0.80, 0.81, and 0.87, respectively.

## 5 DISCUSSION

Currently, there is a shortage of large‐scale, multicenter participatory reports regarding the follow‐up of symptoms after acute‐phase depression treatment in China. The absence of clinical studies has resulted in limited knowledge about the symptoms experienced by depressed patients in China following acute‐phase treatment. This was a nationwide cross‐sectional, multisite follow‐up project focusing on the clinical outcomes of depressive patients in China. After acute treatment, this study conducted a 6‐month follow‐up survey of depressed patients. Key indicators included patients' gender, age, marital status, education level, personal income, underlying illnesses, and history of depression detection and treatment. Patients' medication usage was also summarized, and follow‐up assessments were performed at 3 and 6 months, respectively. The results revealed significant differences between the > 45 years and ≤45 years groups regarding gender, marital status, income, age at first presentation, physical illnesses, and use of antipsychotic and benzodiazepine combinations. Furthermore, statistical differences existed between these groups across three self‐rating scales: QIDS‐SR16 score, PHQ15 score, and SDS score (total score, work/study, social life, and family responsibility) (all *p* < .05). Epidemiological surveys indicate that the discrepancy in depression incidence between men and women starts to emerge specifically during adolescence, typically between the ages of 11 and 14 years (Rydberg Sterner et al., 2020). The hypothesis suggests that the variance in depression incidence between men and women might be linked to the secretion of sex hormones during adolescence. Typically, the gender ratio tends to be close to 1:2 in these cases (Fredrick & Demaray, 2018; Rowniak et al., 2019). Similarly, age acts as a limiting factor influencing the onset of depression. Research conducted de la Torre et al. (2021) within a British population revealed that the likelihood of potential depression was notably higher among individuals aged 45–59 years compared to those aged 16–29 years. In this study, the male‐to‐female ratio was more pronounced than 1:2 (85/138) in the group aged ≤45 years; however, in the group aged > 45 years, it was less than 1:2 (51/154). The findings indicated a progressive pattern of heightened likelihood of depression in women as age increased, while this likelihood decreased among male patients. This trend aligns with the outcomes observed in our phase I study regarding residual symptoms of depression (Zhao et al., 2017). Variations in hormone levels during the physiological cycle, pregnancy, perimenopause, and menopause potentially contribute to the onset and progression of depression in women (Saraswat et al., 2021). Apart from the previously mentioned factors, the rise in depression among the older age group might also stem from the general lack of economic autonomy traditionally experienced by Chinese women, leading to lower social status and bearing heavier family burdens. Conversely, with the economic reforms in China over the last 40 years, women have gained increased independence, correlating with a declining trend in depression.

The current research increasingly indicates a close relationship between marital status and the occurrence of mental illness (Nahar et al., 2020). In this study, the percentage of single individuals in the age group ≤45 years (76/223) was higher compared to those in the age group > 45 years (1/206), showing a significant difference between the two groups (*p* < .05). Additionally, within the ≤45 years group, the proportion of single individuals with higher education surpassed those with lower education (42.1% vs. 19.5%), demonstrating a significant difference between these groups (*p* < .05). This trend could be associated with the higher education level of this generation and the immense social and occupational pressures they face, leading to feelings of helplessness, disappointment, frustration, and loneliness. Conversely, among individuals in the >45 years group, the proportion of married patients was higher (181/205). Many longitudinal and cross‐sectional studies have shown that poor marital status is often a risk factor for depression in middle‐aged and older adults (Maier et al., 2021; Richardson et al., 2020). Williams and Umberson (2004) proposed the Marital Resource Model and the Crisis Model as theoretical explanations for the causal relationship between marital status and depressive symptoms from a theoretical perspective. Within this study, the age of initial depression onset was notably lower in more educated patients compared to those with less education, regardless of the age group. This discrepancy might be attributed to individuals with higher education possessing earlier and more comprehensive knowledge about depression, leading to earlier hospital admissions following the onset of depression.

Regarding medication usage, the utilization of a combination of antipsychotics and benzodiazepines was significantly lower in patients aged ≤45 years compared to those in the >45 years age group (*p* < .05). Among young adults, individuals with higher education levels used benzodiazepines significantly more than those with lower education (*p* < .05). However, among middle‐aged and older adults, education did not influence the use of benzodiazepines versus antipsychotics. This difference might be attributed to the increased usage of antipsychotics and benzodiazepines in middle‐aged and older adults, possibly due to age‐related increases in organic psychotic symptoms associated with insomnia.

Conversely, anxiety and insomnia were more prevalent in young individuals with higher education compared to those without higher education, resulting in an increased utilization of benzodiazepines. The QIDS‐SR16 score, the PHQ15 score, and the SDS scale are all reliable indicators in assessing patients with depression (Dell'Osso et al., 2015; Zu et al., 2021). The three indicators evaluate patients' residual symptoms, severity, somatic symptoms, and social functioning. In this study, following 2–3 months of acute‐phase treatment, patients aged over 45 years exhibited notably lower QIDS‐SR16 scores, PHQ15 scores, all SDS scores, and residual symptoms compared to the group aged 45 years or younger (all *p* < .05, respectively). After treatment in the acute phase, middle‐aged and older patients had significantly fewer residual symptoms of depression and somatic symptoms and minor impairment in social functioning than younger patients. Previous studies have indicated that depressive symptoms tend to become more pronounced as patients age. Saragih et al.’s (2021) study of healthcare workers during the COVID‐19 pandemic revealed an increase in depressive symptoms with age. However, their findings also highlighted that depressive symptoms were most pronounced among individuals aged 29–35 years. In the current study, middle‐aged and older patients exhibited fewer residual symptoms of depression and experienced minor impairment in social functioning during the consolidation period compared to younger patients. This difference could be attributed to the wealth of life experiences and adopting more flexible and diverse coping strategies in this age group when dealing with life challenges. Older adults might opt for emotion‐focused coping mechanisms to manage difficulties, including pain and limitations in activity (Nakagawa et al., 2013). In this study, patients over 45 primarily belong to the cohort born before China's economic reforms. This demographic has encountered more life and ideological stress than those born after 1980. The accumulation of life experiences has equipped them with stronger willpower and coping abilities to manage emotions associated with depression when facing setbacks. Another potential factor is that the younger group confronts heightened workplace and life stress in contemporary society, where the persistent presence of stressors might impede the elimination of residual symptoms and hinder the recovery of social functioning. The most prominent depressive symptoms were observed within the age brackets of 25–35 (Lu et al., 2021; Rezaei et al., 2022; Saragih et al., 2021; Xu et al., 2021) and the “U” shaped correlation between happiness and age suggests that young individuals tend to experience heightened depression when confronted with stress (Steptoe et al., 2015; Xu et al., 2019).

There is no consensus regarding the impact of education level on depressive symptoms. Some studies have indicated that depressive symptoms tend to be more pronounced among individuals with higher levels of education (Fukuhara et al., 2021; Pei et al., 2020). Indeed, certain studies have concluded that individuals with higher levels of education exhibit elevated levels of mental health (Xu et al., 2021). However, the safeguarding impact of education against symptoms of depression seemed to diminish under the influence of the COVID‐19 pandemic (Gigantesco et al., 2022). Indeed, some studies have also concluded that the level of education does not influence people's depressive symptoms (Kim & Park, 2021). The above findings suggest the complexity of the impact of education on depressive symptoms. Further clinical trials are required to thoroughly investigate the effects of varying educational levels on depressive symptoms. Previous studies have seldom analyzed the correlation between residual symptoms post‐acute phase treatment. We specifically examined residual symptoms of depression, residual somatic symptoms, and social functioning in patients with differing educational backgrounds within the age groups of ≤45 years and >45 years, respectively. Patients with varying education levels in both age categories exhibited no statistically significant differences in QIDS‐SR16 total score, PHQ15 score, and SDS indicators at various follow‐up periods—baseline, 3 months, and 6 months (all *p* > .05). The protective theory posits that individuals with higher levels of education possess additional skills and strategies to effectively manage and cope with depression (Shen, 2020). Different theories propose that individuals with lower educational attainment might take longer to navigate and manage economic and employment‐related stress than those with higher educational attainment. As a result, this group may exhibit higher resilience to stressors over time (Luo et al., 2021). The second potential reason is that individuals with higher education levels and depression might have acquired more coping skills to manage depressive symptoms. However, they might also be more attuned and sensitive to their symptoms, resulting in a higher likelihood of reporting them. Both aspects suggest that within the population aged 45 or younger, the 6‐month follow‐up indicates notably higher levels of depression among patients with lower educational levels compared to those with higher educational levels. Simultaneously, there exists no significant difference in the overall scores.

Furthermore, individuals with higher education tend to prioritize the pursuit of self‐actualization. When these needs for self‐fulfillment remain unmet, symptoms of depression can persist for extended periods. This rationale aligns with the observed trend of significantly higher depression rates in developed countries compared to developing ones (Yusuf et al., 2020).

## 6 CONCLUSION

Age affects the prognosis of depressed patients after acute treatment. Patients with depression younger than 45 years of age have more residual depressive symptoms, more somatic symptoms, and more severe impairment in social functioning. There was no significant difference in the prognosis of patients in different educational attainment groups of different ages in the following period. In this study, we conducted a comprehensive analysis of the treatment and recovery outcomes of depression among patients of different ages and educational backgrounds in China, which provides us with a starting point for further research on depression. Further clinical studies can be conducted for depressed patients <45 years of age with residual symptoms and slower recovery of social functioning in order to develop a more targeted treatment plan. Social support such as community services, psychological counseling, and rehabilitation skills training should be provided for patients <45 years of age and enhancing medication regimens.

## AUTHOR CONTRIBUTIONS

Si Zu contributed to the study design, data collection, and statistical analysis, and drafted the initial manuscript while revising it for intellectual content. Dong Wang assisted with data collection and statistical analyses, contributing to the interpretation of results and manuscript revision. Jiexin Fang participated in study design and data collection, collaborating on the analysis and interpretation of findings. Le Xiao was involved in data collection and the management of patient follow‐up, assisting in manuscript preparation and revisions. Xuequan Zhu coordinated the study across multiple centers and contributed to data analysis. Wenyuan Wu aided in the development of the study protocol and provided critical revisions to the manuscript. Xiufeng Lin offered expertise in the assessment tools utilized in the study and contributed to results interpretation. Gang Wang led the study design and coordination, overseeing data analysis, interpretation of findings, and providing critical revisions to the manuscript. Yongdong Hu contributed to the study design and methodology, assisting in data interpretation and manuscript preparation. All authors approved the final version of the manuscript and agree to be accountable for all aspects of the work.

## CONFLICT OF INTEREST STATEMENT

Author Xiufeng Lin is employed by 3S‐Family (Beijing) Science & Technologies Co Ltd, Beijing, China. The remaining authors declare that the research was conducted in the absence of any commercial or financial relationships that could be construed as a potential conflict of interest.

## FUNDING INFORMATION

No funding was received for this study.

### PEER REVIEW

The peer review history for this article is available at https://publons.com/publon/10.1002/brb3.70024.

## Data Availability

The datasets generated and analyzed during the current study are available from the corresponding author on reasonable request.
